# The first draft genomes of the ant *Formica exsecta,* and its *Wolbachia* endosymbiont reveal extensive gene transfer from endosymbiont to host

**DOI:** 10.1186/s12864-019-5665-6

**Published:** 2019-04-16

**Authors:** Kishor Dhaygude, Abhilash Nair, Helena Johansson, Yannick Wurm, Liselotte Sundström

**Affiliations:** 1 Organismal and Evolutionary Biology Research Programme, Faculty of Biological and environmental sciences, University of Helsinki, P.O. Box 65, FI-00014 Helsinki, Finland; 2 Organismal Biology Department, School of Biological and Chemical Sciences, Queen Mary University of London, Mile End Road, London, E1 4NS UK; 3 Tvärminne Zoological Station, University of Helsinki, J.A. Palménin tie 260, FI-10900 Hanko, Finland

**Keywords:** *Formica exsecta*, Genome, Endosymbionts, Transposons, Horizontal gene transfer, *Wolbachia*

## Abstract

**Background:**

Adapting to changes in the environment is the foundation of species survival, and is usually thought to be a gradual process. However, transposable elements (TEs), epigenetic modifications, and/or genetic material acquired from other organisms by means of horizontal gene transfer (HGTs), can also lead to novel adaptive traits. Social insects form dense societies, which attract and maintain extra- and intracellular accessory inhabitants, which may facilitate gene transfer between species. The wood ant *Formica exsecta* (Formicidae; Hymenoptera), is a common ant species throughout the Palearctic region. The species is a well-established model for studies of ecological characteristics and evolutionary conflict.

**Results:**

In this study, we sequenced and assembled draft genomes for *F. exsecta* and its endosymbiont *Wolbachia*. The *F. exsecta* draft genome is 277.7 Mb long; we identify 13,767 protein coding genes, for which we provide gene ontology and protein domain annotations. This is also the first report of a *Wolbachia* genome from ants, and provides insights into the phylogenetic position of this endosymbiont. We also identified multiple horizontal gene transfer events (HGTs) from *Wolbachia* to *F. exsecta*. Some of these HGTs have also occurred in parallel in multiple other insect genomes, highlighting the extent of HGTs in eukaryotes.

**Conclusion:**

We present the first draft genome of ant *F. exsecta*, and its endosymbiont *Wolbachia* (*w*Fex), and show considerable rates of gene transfer from the symbiont to the host. We expect that especially the *F. exsecta* genome will be valuable resource in further exploration of the molecular basis of the evolution of social organization.

**Electronic supplementary material:**

The online version of this article (10.1186/s12864-019-5665-6) contains supplementary material, which is available to authorized users.

## Background

Adapting to changes in the environment is the foundation of species survival, and is usually thought to be a gradual process. Genomic changes, such as single nucleotide substitutions play key roles in adaptive evolution, although few mutations are beneficial. Besides nucleotide substitutions, other structural and regulatory units, such as transposable elements (TEs) and epigenetic modifications, can also act as drivers in adaptation [1–3]. Genetic material can also be acquired from other organisms by means of horizontal gene transfer (HGTs), and this can also lead to novel adaptive traits [4, 5]. Both mutations and HGTs can drive rapid genome evolution [6, 7]. Horizontal gene transfers have been reported in many taxa, most commonly from bacteria to animals [7], plants [8, 9], fungi [10–12], but the mechanisms that underpin horizontal gene transfer events, and the mode by which bacterial genetic material is integrated into the eukaryote genome are not well understood.

Many cases of horizontal gene transfer from bacteria to eukaryotes involve intracellular endosymbionts, which are maternally transmitted through oocytes [13, 14]. The most common examples of endosymbiont to host horizontal gene transfers involve the bacterium *Wolbachia*, a well described intracellular, maternally inherited gram-negative bacterium known to infect over 60% of the investigated insect species [15–17]. *Wolbachia* infection is also prevalent in filarial nematodes, crustaceans, and arachnids [18–20]. *Wolbachia-* host interactions can be mutualistic or pathogenic [21]. A number of ecdysozoan genomes have been reported to contain chromosomal insertions originating from *Wolbachia*, including the mosquito *Aedes aegypti* [22, 23], the longhorn beetle *Monochamus alternatus* [24], filarial nematodes of the genera *Onchocerca, Brugia*, and *Dirofilaria* [20, 25], parasitoid wasps of the genus *Nasonia*, the fruit fly *Drosophila ananassae*, the pea aphid *Acyrthosiphon pisum* [26, 27], and the bean beetle *Callosobruchus chinensis* [28]. Although most of the transferred DNA is probably nonfunctional in the host genome [25, 28, 29], some of the transferred genes are functional [22]. The functional HGT events can be categorized into two broad types – one that maintains pre-existing functions in the recipient host, and one that provides the recipient host with new functionality, including altered host nutrition, protection and adaptation to extreme environments [30].

Infection with *Wolbachia* is widespread in Hymenoptera. Most hymenopteran *Wolbachia* infections have the cytoplasmic incompatibility phenotype [31], which leads to reproductive incompatibility between infected sperm and uninfected eggs. The ants that have been investigated so far are infected with A-group strains of CI-inducing *Wolbachia* [32–34]. Wenseleers et al. [35] showed that 25 out of 50 species of ants in Java and Sumatra screened positive for a single A-group strain of *Wolbachia*. By contrast, a study on a single Swiss population of the ant *Formica exsecta*, found that all the ants tested were infected with four or five different strains of *Wolbachia* [32, 36].

The aims of this study are to produce the first genome for the ant genus *Formica*, to test whether horizontally transferred genetic elements exist in the genome of the ant *F. exsecta*, and to describe the genomic organization of any such elements. The genus *Formica* is listed by the Global Ant Genome Alliance (GAGA) as one of the high-priority ant taxons to be sequenced [37], owing to its key taxonomic position, and the ecological and behavioral data that are available for the species. We report the first whole genome sequencing of this species, and the draft genome sequence of its associated cytoplasmic *Wolbachia* endosymbiont (*w*Fex). We further report the presence of multiple extensive insertions of *Wolbachia* genetic material in the host genome, and compare the HGTs insertions discovered in the assembled draft genome to other genomes, to understand the pattern of HGT events between endosymbiont and host. We analyze in detail the genomic features of *F. exsecta* along with its endosymbiont *Wolbachia,* and discuss our findings in the light of genome evolution in *Wolbachia* and its host.

## Results & discussion

### The *F. exsecta* genome

**Fig. 1:**
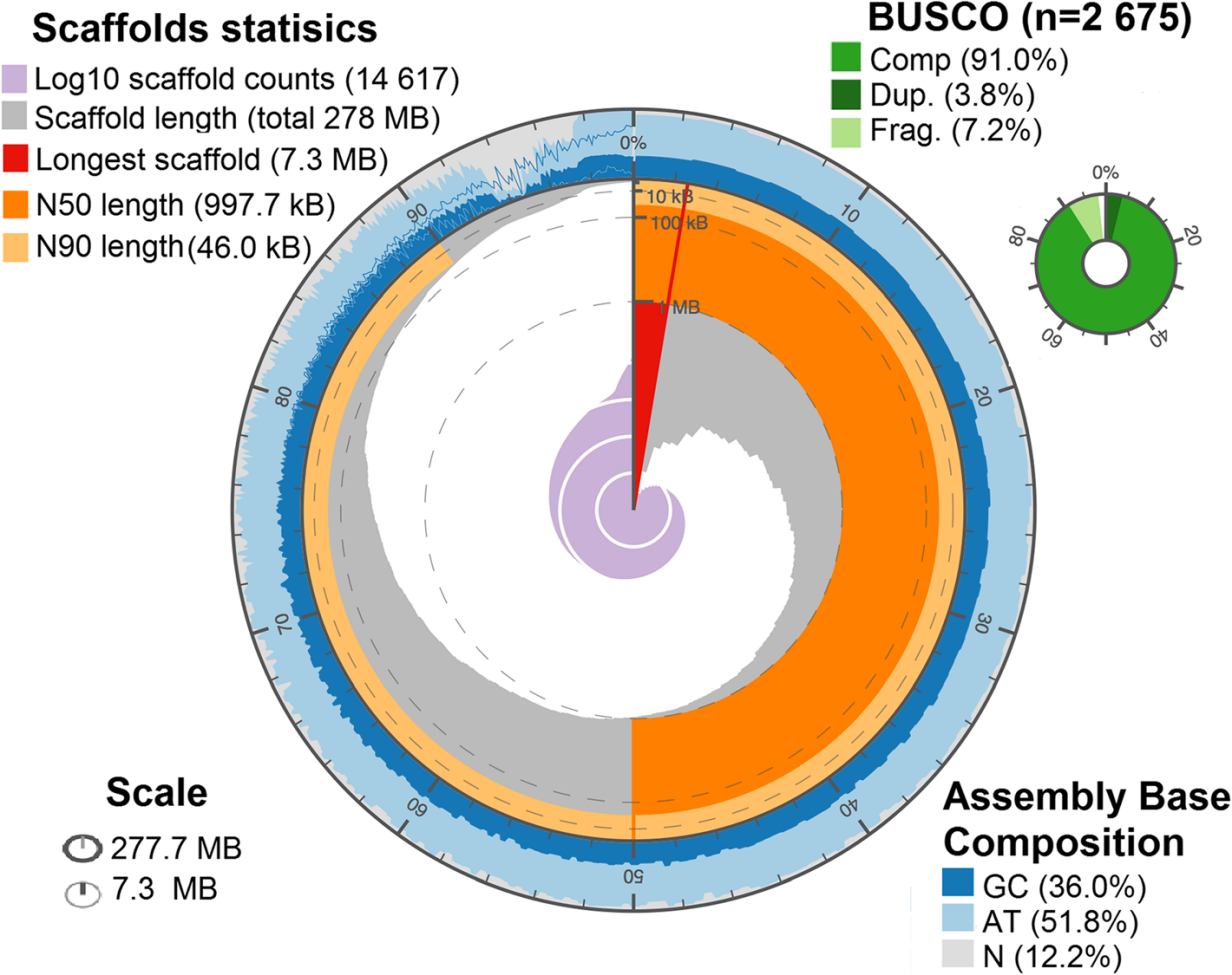
De novo genome assembly of *F. exsecta* genome, summarized by the following metrics: **a**) Overall assembly length, **b**) Number of scaffolds/contigs, **c**) Length of the longest scaffold/contig, **d**) Scaffold/contig N50 and N90, **e**) Percentage GCs and percentage Ns, f) BUSCO completeness, g) Scaffold/contig length/count distribution

The Illumina sequencing libraries from DNA extracted from testes of males of a *F. exsecta* colony yielded > 99 gigabases of Illumina sequence data. The final genome resulting from the assembly of these data was 277.7 megabases (Mb) long, encompassing 14,617 scaffolds (Fig. 1) with a N50 scaffold length of 997.7 kb (Table 1). The number of scaffolds is higher than the number of chromosomes (*n* = 26) reported for *F. exsecta* [38, 39]. Similarly, the *F. exsecta* genome assembly is somewhat shorter than genome size estimates obtained by flow cytometry for species in the subfamily Formicinae (range: 296–385 Mb) [40]. These discrepancies are unsurprising given the difficulty of assembling highly repetitive gene content from short sequencing reads [41]. In line with this, the genome assembly length metrics are similar to those of the 23 ant genomes that have been published. The raw data, gapped scaffolds, and annotations underpinning this assembly are deposited on public databases under BioProject PRJNA393850 (accession NPMM00000000).

**Table 1:** Genome assembly statistics for *F. exsecta* and its *Wolbachia* endosymbiont

Genome Assembly Stats	*Formica exsecta* Genome	FE *Wolbachia* endosymbiont Genome
Total length	277,719,392 (277 MB)	3,096,460 (3.09 MB)
Total contigs	14,617	69
Contigs (> = 1000 bp)	3136 (98.24% genome)	68 (99.97% genome)
Contigs (> = 50,000 bp)	545 (89.59% genome)	22 (75.48% genome)
N50:	997,654 bp	104,167 bp
N75:	318,356 bp	54,296 bp
L50:	73	11
L75:	185	22
GC (%)	36.00	35.13

### Quantitative assessment of genome assembly

Based on scaffold N50 and N75 statistics, contig size, and GC content, the *F. exsecta* genome assembly is comparable in quality and completeness to other sequenced ant genomes (Additional file 1: Table S1). All the 248 CEGMA eukaryotic core genes were found, and 241 of these genes were complete in length. Similarly, 98.5% of 1634 BUSCO Insecta genes were complete in the genome (Table 2). These results held with other BUSCO analysis levels including Eukaryota, Arthropoda, and Hymenoptera, with low duplication levels (2.2 to 5.3%), and a few missing genes (0.6 to 1.27%; Table 2). Such discrepancies can be due to technical artifacts such as sequencing biases or assembly difficulties, as well as to true differences between our *F. exsecta* sample and the BUSCO and CEGMA datasets. To further evaluate genome completeness, we compared the independently generated *F. exsecta* transcriptome [42] to the genome reported here. More than 98.75% of the 10.999 assembled ESTs mapped unambiguously to the genome (BLASTn E < 10^− 50^). Together, these analyses show that the genome assembly has high completeness.

**Table 2:** BUSCO quality metrics for the genome assemblies of *F. exsecta* and the *Wolbachia* endosymbiont *of F. exsecta (wFex)*

	*Formica exsecta* Genome				*wFex* Genome	
BUSCO metric	Eukaryota	Insecta	Arthropoda	Hymenoptera	Bacteria	Proteobacteria
Complete	299 (98.7%)	1634 (98.5%)	2549 (95.29%)	4249 (96.2%)	107 (72.30%)	158 (71.49%)
Complete and single copy	283 (93.4%)	1572 (94.8%)	2446 (91.44%)	4151 (94.0%)	35 (23.65%)	55 (24.88)
Complete and duplicated	16 (5.3%)	62 (3.7%)	103 (3.86%)	98 (2.2%)	72 (48.65%)	103 (46.60%)
Fragmented	1 (0.3%)	15 (0.9%)	195 (7.29%)	123 (2.8%)	9 (6.08%)	11 (4.97%)
Missing	3 (1.0%)	9 (0.6%)	34 (1.27%)	43 (1.0%)	32 (21.62%)	52 (23.52%)
Total	303 (100%)	1658 (100%)	2675 (100%)	4415 (100%)	148 (100%)	221 (100%)

### Gene content in the *F. exsecta* genome

We identified 13,637 protein coding genes by combining ab initio, EST-based, and sequence similarity based gene predictions methods. The GC content was higher in exons (41.6%) than in introns (30.6%), a pattern similar to that reported in the honey bee, *Apis mellifera*, and the fire ant, *Solenopsis invicta* [43, 44]. Despite this, as in other ant genomes [37, 45], the overall GC content in genes (35.1%) was similar to the rest of the genome (36.0%).

**Fig. 2:**
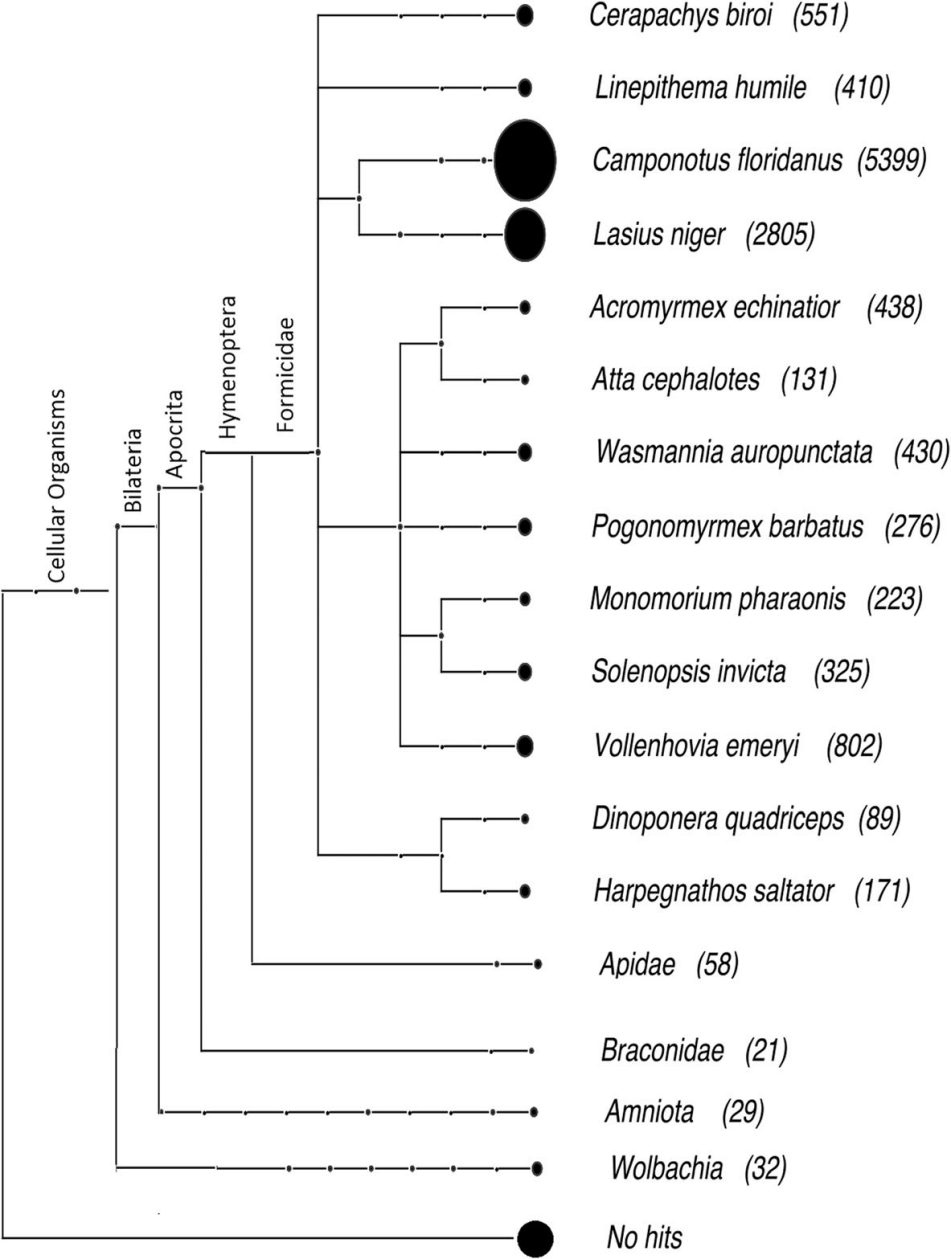
Taxonomic distribution of the best BLASTp hits of *F. exsecta* proteins to the non-redundant (nr) protein database (E < 10^− 5^)

We used Basic Local Alignment Search Tool (BLAST) and orthology analyses to characterize *F. exsecta* genes. The vast majority (88%; 12,050) of these had the highest BLASTp similarity to genes in other ants. A further 0.4% had the highest similarity to Apidae, and 0.6% to Braconidae, Amniota, and *Wolbachia* (the latter probably due to HGT; see below and Fig. 2). The remaining 3.09% belong to other taxa not included in Fig. 2 because they had fewer than 20 hits. The remaining genes (7.91%, *n* = 1080) lacked clear sequence similarity [cutoff for BLASTx E < 10^− 3^] to known protein sequences or protein domains. Some of these may represent erroneous gene predictions [46], however 994 of them are ≥1000 bp, and include an open reading frame > 300 amino acids long, which is unlikely to occur by chance. Importantly, although only a single pooled transcriptome library, prepared from different developmental life stage samples, was available for *F. exsecta*, 235 of the genes are expressed (FPKM ≥1) [42]. It is thus likely that a high proportion of the 1080 genes (7.91%) are taxonomically restricted genes, unique to the *F. exsecta* lineage. Information on taxonomically restricted genes in the other published Formicinae genomes (*Camponotus floridanus*, *Lasius niger*, *Formica selysi*) is limited, but the initial publication of the genome of *Solenopsis invicta* (Myrmicinae) reported 18% taxonomically restricted genes [43], and a study comparing genomes of 7 ant species found an average of 1715 species-specific genes [47], indicating that high proportions of taxon-specific genes can be present also in other ant.

**Fig. 3:**
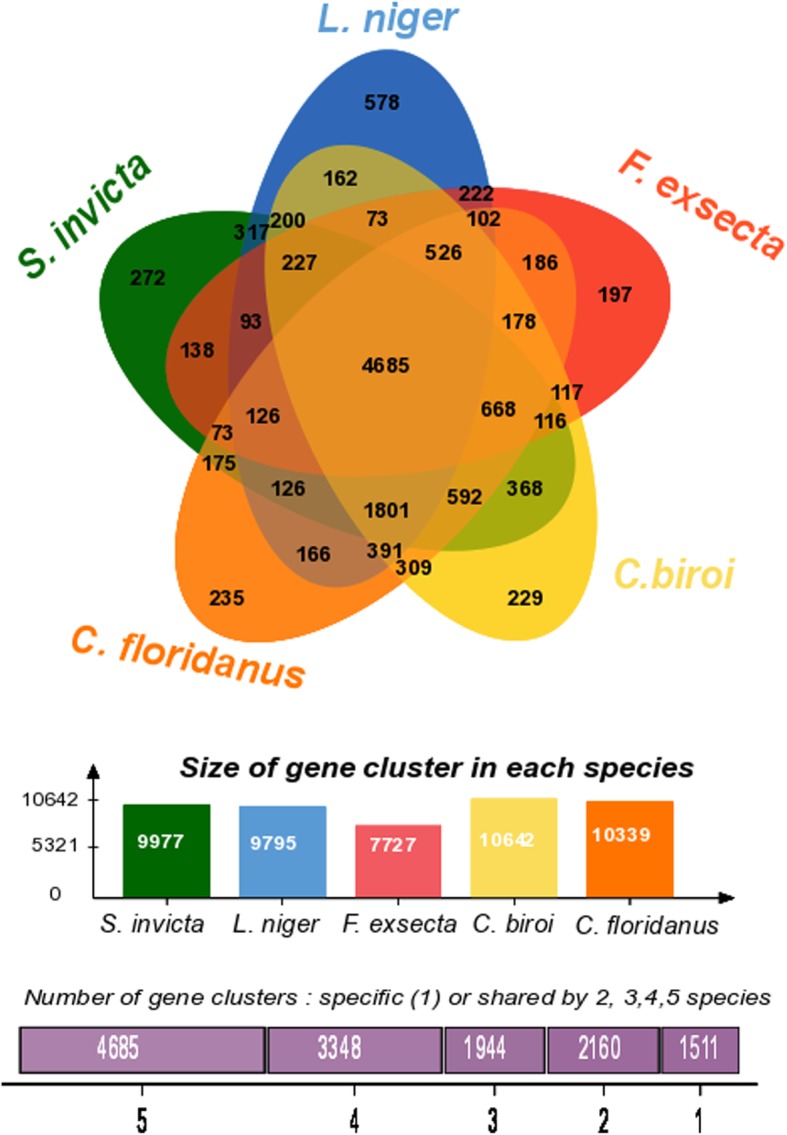
Venn diagram showing the distribution of gene families (orthologous clusters) among five ant species including three closely related members of the subfamily Formicinae (*F. exsecta, Camponotus floridanus, Lasius niger*)*,* and two distinctly related ants *(Solenopsis invicta* and *Cerapachys biroi)*

The genes of *F. exsecta* (*n* = 13,637) were grouped into 7727 orthologous clusters (Fig. 3). Comparative analysis of the *F. exsecta* genes with the closely related species *Camponotus floridanus* and *Lasius niger*, and the more distantly related *Solenopsis invicta* and *Cerapachys biroi* revealed that 4685 out of 7727 orthologous clusters are shared between all five species. In addition, we found 102 gene clusters that were exclusive to three Formicinae genomes (*F. exsecta*, *Camponotus floridanus* and *Lasius niger;* Additional file 2: Table S2). Such genes are important candidates that could be involved in the evolution of this subfamily. Many of the genes in these clusters had no detectable relation to existing genes outside the Formicinae; those that did, included GO annotations such as glycerate kinase, transferase activity, deoxyribonucleoside diphosphate metabolic process.

Interestingly, 633 of the *F. exsecta*-specific genes could be grouped into 197 ortholog clusters of 2 or more genes (Additional file 3: Table S3), suggesting not only newly evolved genes, but also potential gene duplication and subfunctionalisation. Previous comparative genome studies have indicated that 10–20% of genes lack recognizable homologs in other species in every taxonomic group so far studied [48–51]. Our lower percentage of orphan genes could be due to our hierarchical approach to annotation, the wide range of databases used, and the large amounts of ant genomic data generated over the past years [52].

### Genes with signatures of evolution under positive selection

We performed analyses to detect genes with signatures of positive selection in *F. exsecta.* First, selection analysis (dN/dS ratio estimations) on 3157 single-copy genes shared between the five core ant species (without paralogous genes), revealed that 500 genes have signatures of positive selection in the lineage leading to *F. exsecta*. These include genes involved in fatty acid metabolism, lipid catabolism, and chitin metabolism (Additional file 4: Table S4). Interestingly, previous studies on ants, bees, and flies also provide evidence for positive selection on genes in similar functional categories as in our study [53]. For example, genes involved in biological functions such as carbohydrate metabolic processes, lipid metabolic processes, cytoskeleton organization, cell surface receptor signaling pathways, and RNA processing were overrepresented in the enrichment analysis, and such genes were also previously reported as positively selected genes in ants, bees, and flies [53, 54].

To perform a similar analysis on a larger number of genes, we used a second approach based on pairwise comparisons between *F. exsecta* and *Camponotus floridanus*. Out of 5148 one-to-one- orthologs, 29 showed dN/dS > 1 (*P* < 0.005; Additional file 5: Table S5). Although some of these putative genes could be artefactual or non-coding, they all include an open reading frame of > 100 amino acids. Five (17%) out of 29 genes are likely linked to transposon activity as they are transposase-like or have EpsG domains. Among the other genes, only a few are annotated: the Icarapin-like protein is a venom gene, and such genes have been shown to be under positive selection in wasps [55]. Perhaps more surprisingly we found a high dN/dS ratio for the Homeobox protein gene orthopedia, which is involved in early embryonic development [56]. The orthopedia gene plays a significant role in the development of the nervous system in both fruit flies (*Drosophila sp.*)*,* and mice (*Mus musculus*) [57], and has both novel and conserved roles in other taxa [58]. The diversification of this gene could contribute to the evolution of the nervous system in these ants. The modalities of the putative faster evolution of this gene will become clearer as further transcriptomic data becomes available from *F. execta,* and genome sequence becomes available from other Formicinae.

### Repetitive elements

Repetitive elements comprised 15.88% (44.10 Mb) of the *F. exsecta* assembly. This proportion is similar to that found in other ants (16.5–31.5% [45]. This is probably an underestimate because (i) genomic regions that cannot be assembled are enriched with such repeats, (ii) multiple copies of a repetitive element are often collapsed into a single copy during genome assembly, and (iii) only a portion of repetitive elements in *F. exsecta* will have similarity to sequences in standard repeat databases. Overall, 3.18% (8.8 Mb) of the assembly was composed of simple repeats, whereas 12.73% (35.34 Mb) comprised interspersed repeats, most of which (53.73%) could not be classified. Among those that could be classified, 10,542 retro element fragments represented 2.74% of the genome, and 53,438 DNA transposons represented 4.23% of the genome. The *F. exsecta* genome contains copies of the piggyBac transposon (23 in total, and 7 within intact ORFs). Higher numbers (234) of piggyBac transposons have been found in *Camponotus floridanus,* yet only 6 of these were found within ORFs [59].

### The *Wolbachia* endosymbiont genome of *F. exsecta*

The assembly of the “*Wolbachia* endosymbiont genome of *F. exsecta*” (henceforth *w*Fex), was 3.09 Mb long, encompassing 69 scaffolds with a N50 scaffold length of 104,167 nt, and a GC content of 35.13% (Table 1; GenBank, Bioproject: PRJNA436771). This assembly of *w*Fex shows extensive nucleotide similarity with the complete genome of the *Wolbachia* endosymbiont of *Drosophila simulans*, wRi (GenBank ID: NC_012416.1), and the *Wolbachia* endosymbiont of *Dactylopius coccus*, strain *w*DacA (GenBank ID: NZ_LSYX00000000) (Additional file 6: Figure S1). We determined that 549 genes are present as a single copy in the *Wolbachia* genomes most closely related to *w*Fex ([60] see below); 537 (99.6%) out of these 539 core genes are present in the *w*Fex genome, suggesting high completeness.

However, the *w*Fex genome is considerably larger (3.09 Mb) than the *Wolbachia* genomes reported previously (range: 0.95 to 1.66 Mb) [61], and includes a greater number of open reading frames (1796 ORFs) than other published *Wolbachia* genomes [range: 644 to 1275 genes]. *Formica exsecta* is known to carry more than one *Wolbachia* strain [36], thus these patterns could be due to the presence of multiple endosymbiont strains. Three lines of evidence provide support for this. First, 212 genes (11.80%), which are present as single-copy genes in the *w*Mel, *w*Ri, and *w*Dac genomes [62–64], are present twice in our assembly (Additional file 7: Table S6). Conversely, the *Wolbachia* strains are apparently very closely related and thus have highly similar genomic regions which were collapsed during assembly, which may explain why only 11.8% of the single copy genes differ. Second, 92 (12%) of the 775 genes present as single copies in *w*Fex, included genetic variation within our sample. This also included the cytochrome c oxidase subunit I (*CoxA*), where no such variation is normally expected. Finally, we found 2 copies of the MLST genes (*ftsZ*, *hcpA* and *gatB*), and of the CI inducing genes *cifA* and *cifB* [65], on different scaffolds. Thus it is highly likely that the assemby of *w*Fex comprises multiple strains. Despite extensive attempts, we were unable to disentangle the two or more *Wolbachia* strains, probably because differences in synteny between the strains cannot be resolved using short-read sequence data. Similar assembly artifacts, due to multiple *Wolbachia* strains, have also been reported by other studies [64].

**Fig. 4:**
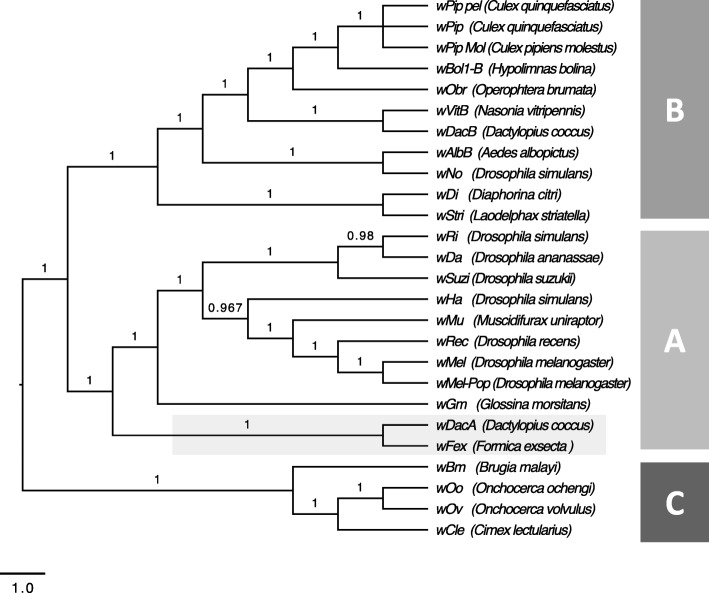
Phylogeny of the *Wolbachia* supergroups **a**, **b**, and **c** strains with the newly assembled *w*Fex genome. The phylogenetic reconstructions are based on individual analyses of 12 single copy core genes of 25 *Wolbachia* strains. The support values on the branch labels indicate Bayesian posterior probabilities. The letters **a**-**c** indicate the separate supergroups

To determine how *w*Fex is related to other *Wolbachia*, we used Bayesian phylogenetic analysis based on 12 single copy genes (Additional file 8: Table S7) from the 25 available *Wolbachia* genomes from the NCBI database. The analysis revealed three distinct monophyletic clades, all with posterior probabilities > 0.9. Each of these clades represent one super group of *Wolbachia* (Fig. 4). Of these three supergroups, two have been found only in arthropods (super groups A and B), whereas the third super group is found only in filarial nematodes (super group C) [17]. In the phylogenetic analysis, *w*Fex clustered with the *Wolbachia* strains within super group A. This is consistent with earlier studies on *Wolbachia* in ants, which also found supergroup A in the majority of the infected ants [31]. The closest relative of *w*Fex was the strain *w*DacA which infects the scale insect, *Dactylopius coccus*. Our phylogeny is also consistent with the recent published phylogeny of *Wolbachia* [66].

**Fig. 5:**
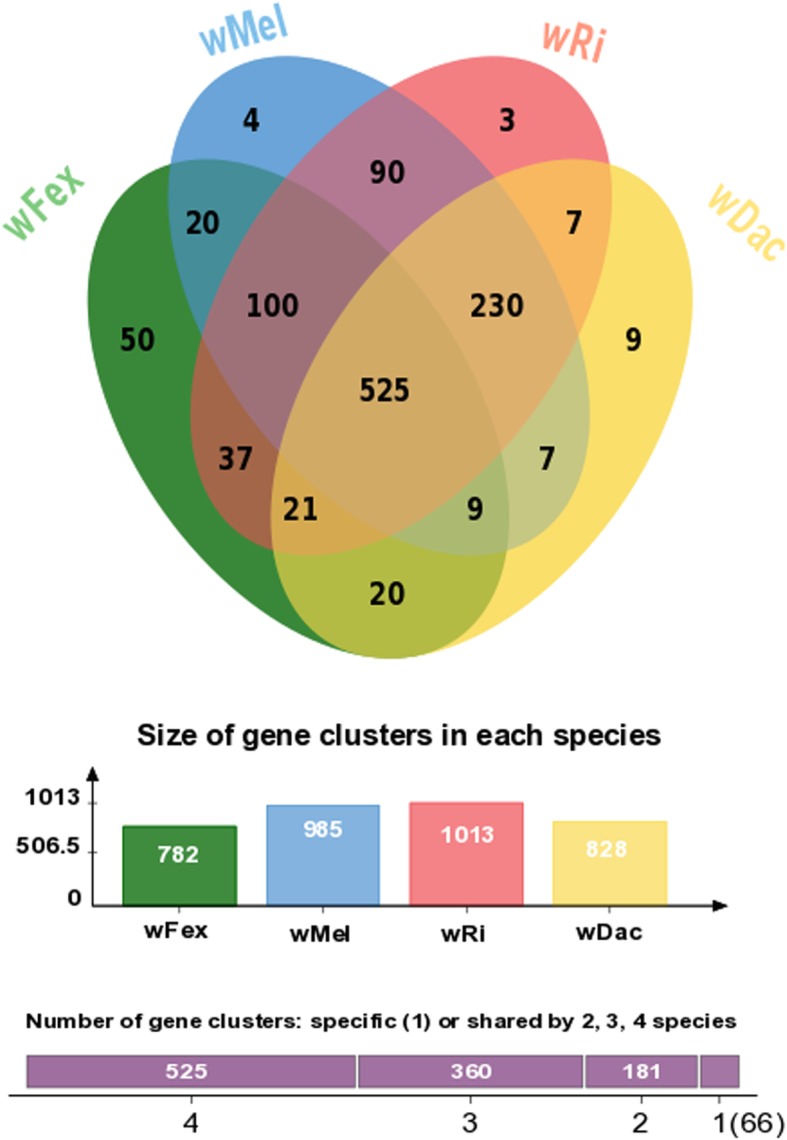
Venn diagram displaying the overlap in orthologous genes among four *Wolbachia* species including the newly assembled *w*Fex strain and the *w*Dac, *w*Ri, *w*Mel strains reported previously

Given that *w*Fex affiliates with the supergroup A in our phylogenetic analysis, we investigated the extent to which its gene content aligned with that of other *Wolbachia* genomes in the same supergroup. The taxonomic distribution of the best BLASTp (protein blast) hits of the *w*Fex protein to the nonredundant protein (nr) database had highest similarity to *w*Dac protein sequences. This supports the inference made from the phylogeny, that *w*Fex is more closely related to *w*Dac than to other *Wolbachia* strains. We found that 525 genes were shared across all strains in this supergroup A, including *w*Fex (Fig. 5). About 20% of these genes had no match to known proteins, whereas the remaining genes matched a wide range of predicted functions [60, 63]. We also found strain-specific genes (*w*Fex - 50 genes, *w*Mel - 4 genes, *w*Ri - 3 genes, *w*Dac - 9 genes). The *w*Fex-specific genes included inferred annotations including Ankyrin repeat protein, ATP synthase, and chromosome partition protein (Additional file 9: Table S8). These strain-specific genes can provide an interesting snapshot of the evolutionary dynamics of a species. For example, ankyrin repeat proteins are involved in numerous functional processes, and have been suggested to play an important role in host-symbiont interactions [67]. Comparative analyses suggest that they may be involved in host communication and reproductive phenotypes [68].

To explore differences in gene content between CI-inducing, and non-CI-inducing strains of *Wolbachia*, homologous genes in six CI-inducing, and three non-CI-inducing strains were aligned, and compared [60]. The non-CI-inducing *Wolbachia* strains (range: 644–805 genes) contained fewer genes than the CI-inducing ones (range: 911–1275 genes). The CI-inducing strains shared 84 genes, not found in the non-CI-inducing strains. We found 80 (95.23%) of these 84 genes in *w*Fex (Additional file 10: Figure S2), as well as the genes *cifA* and *cifB*, which are involved in the CI mechanism (Additional file 11: Figure S3). Both copies of genes appear to be functional as their lengths are 100% in comparison to similar gene sequences available in NCBI database. Together this supports the assumption that *w*Fex is a CI-inducing *Wolbachia* strain, but we warrant that genomic information is unable to conclusively demonstrate this.

### Horizontal gene transfers, and functional novelty

Intracellular symbionts can contribute new genes or fragments of genes to the host genome via horizontal gene transfer [7, 17, 69]. We found evidence for ancestral horizontal transfer of cytoplasmic *Wolbachia* to the host *F. exsecta* in five scaffolds (scaffold83, scaffold233, scaffold574, scaffold707, scaffold741) (chromosomal *Wolbachia*). The four largest transfers are 13 to 47 kb long, and include 83 putative functional protein coding genes, whereas the fifth and smallest insertion (475 bp) lacks protein coding genes, other than a degenerate *Wolbachia* transposase. This transposase is present in 7 out of 29 published *Wolbachia* genomes. The chromosomal *Wolbachia* showed high similarity to the cytoplasmic *Wolbachia* (88.2–99.2%) (Additional file 12: Figure S4). Of the 83 putative functional protein coding genes from chromosomal *Wolbachia*, we found 38 genes in cytoplasmic *Wolbachia* using BLAST; but the other 45 genes were missing. It is difficult for us to validate with certainty whether these 45 genes were absent in *w*Fex due to the fragmented assembly of the cytoplasmic *Wolbachia* (*w*Fex). Our analysis shows that similar transfer events of this homologous fragment apparently also have occurred from cytoplasmic *Wolbachia* to the genomes of the ants *Vollenhovia emeryi* (gene: LOC105557741), and *Cardiocondyla obscurior* (scaffolds scf7180001101632 and scf7180001108526), as well as the microfilarial nematode *Brugia pahangi*, the Arizona spittle bug *Clastoptera arizona,* and the parasitoid wasp *Diachasma alloeum*.

One-third of invertebrate genomes are thought to contain recent *Wolbachia* gene insertions, ranging in size from short segments (< 600 bp), to nearly the entire genome [17, 25]. Most of these transferred fragments contained transposable elements, as well as some other functional genes from the *Wolbachia* genome. The presumptive HGT events from *Wolbachia* to *F. exsecta* are located in or near regions with transposases. Our BLAST results suggest that four of the insert regions had *Wolbachia* transposases, whereas one insert region has a transposase of ant origin. Whether the presence of such transposases close to HGT sites facilitates insertions is unknown. Interestingly, the putative functional protein-coding genes of *Wolbachia* inserted in the *F. exsecta* genome are similar to the genes reported in similar HGTs events in other insect genomes (eg: ABC transporter, Ankyrin repeat containing protein (Table 3) [70, 71]. This could indicate that some HGT events are either more likely to occur or to be retained for reasons that could be neutral or adaptive to the host or to the endosymbiont. The transcriptome of *F. exsecta* shows that at least 6 out of the 83 genes from the *Wolbachia* HGT regions are transcribed, but with a low FPKM values (range 0.04 to 1.6). These low level transcription trait often observed in bacteria-eukaryote HGTs [7, 25, 29].

**Table 3:** HGT inserts from Wolbachia present in the genome of *F. exsecta* with details of its length and position in the *F. exsecta* genome. The presence of similar insert regions in other eukaryote genomes is also shown

*Wolbachia* gene name	HGT region in *F. exsecta*	Length HGT (bp)	Transposon region near HGT	Transposon Name	Observed in other species	Other Host Species name with position of similar insertion
Transposase	scaffold83: 2271642–2,272,117	475	scaffold83: 2271642–2,272,117	transposase	Complete	*Vollenhovia emeryi (LOC105557741), Cardiocondyla obscurior (genes: scf7180001101632 and scf7180001108526), Diachasma alloeum (LOC107035412), Brugia pahangi (BPAG_contig0001587),*
ABC transporter ATP-binding protein, porphobilinogen deaminase, D-alanine--D-alanine ligase, DNA processing protein DprA, triose-phosphate isomerase	scaffold233: 1712452–1,725,498	13,046	scaffold233: 1714122–1,714,241	transposase	Partial (few gene region)	*Vollenhovia emeryi (NW_011967015.1, NW_011967060.1), Wasmannia auropunctata (scf7180000683207, scf7180000730160),Rhagoletis zephyria (NW_016158779.1), Planococcus citri (KF021963.1), Ctenocephalides felis (KC177865.1)*
DNA repair protein RadC, transposase, DNA ligase, ABC transporter permease, ATP-dependent protease La	scaffold574: 102007–116,197	14,190	scaffold574: 105963–106,483	transposase	Partial (few gene region)	*Vollenhovia emeryi (LOC105557101, NW_011966940.1, NW_011966751.1*), *Monomorium pharaonis (scf7180001140281), Rhagoletis zephyria (LOC108377626), Parasteatoda tepidariorum (LOC107444616, LOC107450900)*
probable carboxypeptidase, type IV secretion system,conjugal transfer protein TrbL, lysyl-tRNA synthetase, UDP-N-acetylmuramoylalanine-D-glutamate ligase	scaffold707: 1–38,814	38,813	scaffold707: 35826–36,154	Mariner Mos1 transposase (Ant origin)	Partial (few gene region)	*Vollenhovia emeryi (NW_011966954.1, NW_011966496*), *Wasmannia auropunctata (scf7180000735528), Brugia pahangi (BPAG_contig0000608, BPAG_scaffold0000225)*
DNA methylase, Ankyrin repeat domain protein, regulatory protein RepA, site-specific recombinase, cytochrome b-like	scaffold741: 1–47,265	47,264	scaffold741: 54020–54,482, scaffold741: 52587–52,910	IS110 family transposase, Integrase	Partial (few gene region)	*Vollenhovia emeryi (LOC105557561, NW_011966954.1, NW_011967060.1, NW_011967015.1)*, *Wasmannia auropunctata (LOC105460331, scf7180000733651), Drosophila ananassae (WD_0580 gene)*

## Conclusions

Here we present the first draft genome of the ant *F. exsecta*, and its *Wolbachia* endosymbiont. This is the first report of a *Wolbachia* genome from ants, and provides insights into its phylogenetic position. We further identified multiple HGT events from *Wolbachia* to *F. exsecta*. Some of these have also occurred in parallel in several other insect genomes, highlighting the extent of HGTs in eukaryotes. We expect that the *F. exsecta* genome will be a valuable resource in understanding the molecular basis of the evolution of social organization in ants: Recent genomic comparisons between *Formica selysi* and *Solenopsis invicta* have shown convergent evolution of a social chromosome, that underpins social organisation in these ants [72]. Additional comparison of these genomic regions with *F. exsecta* could provide valuable insights on the evolution of genomic architectures underlying social organization.

## Methods

### Sample collection and genome sequencing

Our study population of *F. exsecta*, located on the Hanko peninsula, Southwestern Finland, has been monitored since 1994, and data on demography, genetic structure, and ecology are available [73–76]. Based on genetic data on colony kin structure most (97%) of the approximately 200 colonies are known to have a single reproductive queen, mated to one or more (usually two) males [73–76]. We selected one single-queen colony from our study population on the island Furuskär (F162), and collected 200 adult males from this colony. We used males because in Hymenoptera these arise through arrhenotoky [77] and are haploid [78], meaning that a pool of males together are representative of the diploid genome of their mother. DNA extraction was done from testis, which contains sperm cells and organ tissue, to avoid contamination by gut microbiota. We used a Qiagen Genomic-tip 20/G extraction kit according to the manufacturer’s protocol. For Illumina sequencing we constructed three small insert paired-end libraries (insert sizes of 200 bp, 500 bp, 800 bp), and four mate pair (large insert paired-end) libraries (insert sizes of 2 kb, 5 kb, 10 kb and 20 kb), each containing DNA from 15 to 50 pooled males. Libraries were prepared using protocols recommended by the manufacturers. Sequencing was done at the Beijing Genomics Institute (BGI) using HiSeq2000, which produced a total of 99.97 GB of raw data (Table 4).

**Table 4:** Summary statistics for the raw sequencing data, before and after filtering reads. “Coverage depth” was calculated based on the estimated assembled genome size (300 Mb)

Insert Size	Pair reads Length (bp)	Raw		After Filter	
		Total Data (G)	Sequence coverage (X)	Total Data (G)	Sequence coverage (X)
170 bp	100 bp	22.68	45.36	20.96	41.93
500 bp	100 bp	8.54	17.08	7.34	14.69
800 bp	100 bp	8.84	17.69	5.14	10.29
2 kb	100 bp	13.23	26.46	7.05	14.10
5 kb	100 bp	14.51	29.02	4.74	9.49
10 kb	100 bp	11.77	23.53	5.51	11.02
20 kb	100 bp	20.40	40.81	2.91	5.81
Total	–	99.97	199.95	53.66	107.32

### Genome assembly

We assembled the *F. exsecta* genome using SOAPdenovo2 version 2.04 [79] in three main steps. First, a de Bruijn graph was constructed using short length insert library reads with default parameters (k-mer value of 45), to construct the contigs. The initial contig assembly contained 104,190 contigs with an N50 size of 22,328 bp, and total length of 276.23 Mb of sequence, at an average depth of coverage of 47.37×. Second, all individual reads were realigned onto the contigs. Because reads are paired, they can aid with scaffolding: The number of reads supporting the adjacency of each pair of contigs was calculated and weighted by the ratio between consistent and conflicting paired ends. Scaffolds were constructed in a stepwise manner using libraries of increasing sizes from 500 bp insert size paired-end reads up to mate-pair of 5 kb insert size. Eighty thousand four hundred seventy-three contigs could not be placed in scaffolds. These are highly similar repetitive sequences, since the cd-hit-est tool [80] showed that 43% of these contigs clustered together at 80% of the sequence length. Third, sequencing gaps in the scaffolds were closed with the two mate-pair libraries (Insert size 10 kb and 20 kb). Overall, these steps produced an initial assembly with an N50 scaffold length of 949,634 bp, and a total length of 289,843,734 bp with each scaffold longer than 200 bp.

We used blobology v1.0 [81] to generate taxon-annotated GC-coverage (TAGC) plots of scaffolds in the genome assembly, which can help to identify bacterial contamination (Additional file 13: Figure S5). The scaffolds for the TAGC plot were successfully annotated to the taxonomic order based on the best BLAST match to the NCBI nt database [82]. This analysis revealed that 74 scaffolds matched the endosymbiotic bacterium *Wolbachia.* Sixty-nine of these scaffolds were removed as we concluded that they are part of the *Wolbachia* genome (see analysis below), but five contigs were retained in the final assembly for *F. exsecta* as they contained both *Wolbachia* and ant sequences. Following this curation, the final draft genome assembly was 277.7 Mb long with an N50 value of 997,654 bp and 36% Guanine-cytosine (GC) content (Table 1).

### Genome assembly of *Wolbachia*

All 25 published *Wolbachia* genomes were obtained from the NCBI database [82]. We aligned the 74 scaffolds from the initial *F. exsecta* assembly that matched with *Wolbachia* against these genomes using MUMmer 3.23 [83], and inspected the alignments manually. Sixty-nine of the 74 scaffolds matched completely to *Wolbachia* genomic regions. These 69 scaffolds represented 3.09 Mb total, with a N50 value of 104,167 bp, and referred to as “the *Wolbachia* endosymbiont genome of *F. exsecta*” (*w*Fex).

The remaining five scaffolds each contained several interspersed fragments with similarity to *Wolbachia* genomes, whereas other parts of these scaffolds had high similarity to genomes of ants [84]. Furthermore, the sequencing coverage of these scaffolds was similar to the *F. exsecta* scaffolds, rather than to the *Wolbachia* scaffolds. Finally, detailed inspection of these scaffolds in a genome browser showed no change in sequencing depth where we identify the interspersed fragments with similarity to *Wolbachia*, which would be expected for erroneous chimeric assembly [85]. These data thus suggest that fragments of *Wolbachia* were horizontally transferred to the *F. exsecta* genome. To corroborate these results with independent approaches, we re-assembled the raw sequencing data with two additional independent algorithms that we expect would make different types of assembly errors than SOAPdenovo. The first software, Velvet version 1.2.09 [86], is also based on a de Bruijn graph; the second, SGA version 0.10.5 [87] is based on a string graph. Both resulting assemblies confirmed the patterns we had seen, and validate the idea that the five SOAPdenovo scaffolds containing sequence with similarity to both ants, and *Wolbachia* represent horizontal gene transfers from *Wolbachia* to *F. exsecta*. To ensure the robustness of the assembly of 69 scaffolds of the *Wolbachia* genome (*w*Fex), we re-assembled the *w*Fex genome by excluding the reads which mapped to the HGT region of *F. exsecta* genome. Thus, the chromosomal *Wolbachia* should not affect the assembly of the cytoplasmic *Wolbachia*.

We further compared the sequences of the horizontally transferred fragments in the five SOAPdenovo scaffolds against the NCBI (nr/nt) database [82], using BLAST 2.2.27 [88] to determine whether these fragments may have also undergone horizontal gene transfer in other arthropod genomes. We performed analogous searches on ant genomes present in the NCBI, and the Fourmidable databases [84]. When a positive match with any other ant or arthropod genomes was found, the exact location of the insertion was determined, and compared with that of *F. exsecta.* Finally, the five scaffolds were also compared to the *F. exsecta* transcriptome [42], using BLASTn 2.2.27, to assess similarity with expressed sequences.

### Quantitative assessment of genome assemblies

The quality of the genome assembly is crucial, as it defines the quality of all subsequent analyses that are based on the genome sequences. We explored multiple assembly options (data not shown), and used two methods to assess assembly quality and robustness in order to select the highest quality assembly. First, we evaluated genome contiguity (number and length of contigs) using Quast 3.2 [89] to assess whether our newly assembled draft genome is comparable to published ant genomes [52] based on assembly statistics (N50, N90). Second, we used core gene content-based quality assessment using CEGMA 2.4 [90] to ascertain that the 248 most highly conserved eukaryotic proteins are present in our genome assembly. We also compared genes present in our genome assembly to single-copy orthologs across four lineage-specific sets (Eukaryota (303 genes), Insecta (1658 genes), Arthropoda (2675 genes), and Hymenoptera (4415 genes)) using the BUSCO 1.1 [91]. In addition, we compared the *F. exsecta* genome with 13 other ant genomes, *Camponotus floridanus, Atta cephalotes, Acromyrmex echinatior, Cardiocondyla obscurior, Cerapachys biroi, Lasius niger, Linepithema humile, Monomorium pharaonis, Pogonomyrmex barbatus, Vollenhovia emeryi, Wasmannia auropunctata, Harpegnathos saltator,* and *Solenopsis invicta* [84], using BUSCO. We report BUSCO quality metrics for the *F. exsecta* genome (Table 2).

The quality of the *Wolbachia* endosymbiont genome was quantified with a similar approach, where we used BUSCO to examine the presence of Universal Single-Copy Orthologs of the Bacteria (148 genes), and the Proteobacteria (221 genes) lineages (Table 2). We also used BUSCO to compare the *w*Fex genome with four other *Wolbachia* genomes, including the *Wolbachia* endosymbionts of *Drosophila simulans (w*Ri and *w*No*), Culex quinquefasciatus (w*Pip*)*, and *Drosophila melanogaster (w*Mel*).*

### Gene prediction

We combined several publicly available data sets and computational gene prediction tools to establish an Official Gene Set (OGS) for the *F. exsecta* genome. First, we used the MAKER version 2.28 pipeline [92, 93], to derive consensus gene models from Augustus version 3.1.0 [94], SNAP version 2016-07-28 [95], and Exonerate version 2.2.0 [96]. For this MAKER prediction we used as input datasets the *F. exsecta* transcriptome (ESTs) (Bioproject ID: PRJNA213662, [42]), and the proteomes of all available ant species (Uniprot download on 20-04-2015). The longest protein at each genomic locus was retained, resulting in a set of 23,517 gene models. Because samples may have different sets of transcripts, owing to different biological conditions or developmental stages [42], we additionally made a separate transcript-spliced assembly using RNA sequences generated from separate libraries for different life stages [42], using the Tophat version 2.1.0 [97], and Cufflinks version 2.2.1 [98]. The assemblies from the different samples were then merged using cuffmerge [98]. We further obtained separate Augustus version 3.1.0 [94], and Glimmer version 3.02 [99] gene models with default settings (Augustus: --species = fly --genemodel = partial, −-strand = both, Glimmer: +f, +s, −g 60). The gene sets and gene models from MAKER and from other programs were then merged. Redundancy was removed by favoring for each transcript the longest prediction starting with a methionine. If several transcripts had the same length we retained the one which had the best support from the cufflinks transcript assembly. This redundancy removal resulted in a final set of 13,637 protein coding gene models (final OGS), which contained 33,121 transcripts.

### Genome annotation

We analyzed the complete official gene sets (OGS) of *F. exsecta* to identify sequence and functional similarity by comparing with different sequence databases using BLAST. By using a ribosomal database, we were able to annotate both the large (LSU), and the small (SSU) subunit ribosomal RNAs. The remaining gene sequences were used for retrieving functional information from other databases (SwissProt, Pfam, PROSITE, and COG). Gene sequences were considered to be coding if they had a strong unique hit to the SwissProt protein database [100, 101], or appeared to be orthologs of known predicted protein-coding genes from ant species based on TrEMBL (Translation of EMBL nucleotide sequence database). We also assigned putative metabolic pathways, functional classes, enzyme classes, GeneOntology terms, and locus names with the AutoFact tool [102]. To further improve annotation, and for assigning biological function (e.g. gene expression, metabolic pathways), we also did orthologous searches by comparing with other Hymenoptera sequences [84]. To quantify variation in the numbers of protein family members, we performed Pfam (version 24.0) [103] and PROSITE profile [104] analyses on proteins obtained from the *F. exsecta* gene set. Our final annotation included gene sequences with retrieved protein-related names, functional domains, and expression in other organisms along with enzyme commission (EC) numbers, pathway information, Cluster of Orthologous Groups (COG), functional classes, and Gene Ontology terms.

### Orthology and evolutionary rates

Comparative genome-wide analysis of orthologous genes was performed with OrthoVenn [105] to compare the predicted *F. exsecta* protein sequences with those of four other ant species, *Camponotus floridanus*, *Lasius niger*, *Solenopsis invicta,* and *Cerapachys biroi,* all of which were downloaded from their respective public NCBI repositories. The predicted proteins of *F. exsecta* and the other four species were uploaded into the OrthoVenn web server for identification and comparison of orthologous clusters [105]. Following clustering, orthAgogue was used for the identification of putative orthology and inparalogy relationships. To deduce the putative function of each ortholog, the first protein sequence from each cluster was searched against the non-redundant protein database UniProt using BLASTp 2.2.27. Pairwise sequence similarities among protein sequences were determined for all species with a BLASTp 2.2.27 (E-value cut-off of 10^− 5^, and an inflation value of 1.5 for MCL). Finally, an interactive Venn diagram, summary counts, and functional summaries of clusters shared between species were visualized using OrthoVenn.

To identify genes under positive or relaxed purifying selection in *F. exsecta*, we estimated the rates of non-synonymous to synonymous changes for core orthologous genes (3156) from five ant species (*F. exsecta*, *Camponotus floridanus*, *Lasius niger*, *Solenopsis invicta,* and *Cerapachys biroi*). For this we only included orthologous groups with one ortholog for each species (no paralogous genes were included) in the analysis. We extracted coding and protein sequences for 3156 orthologous groups from the respective public NCBI repositories for the species included. We then aligned all protein sequences using Clustal Omega [106], and then converted them to nucleotide sequences with PAL2NAL version 14 [107]. We then ran CODEML version 4.9e [107], using the branch site model with *F. exsecta* as foreground branch, and the other five ant species as background lineages. The Bayes empirical method (Yang et al. 2005) was used to estimate the posterior probabilities, which were then used to identify sites under selection. We additionally estimated pairwise dN/dS ratios for orthologous genes (5148 genes) between *Camponotus floridanus* and *F. exsecta* in CODEML.

We also ran an orthology analysis between the proteins from three *Wolbachia* species published previously (*w*Ri, *w*Dac, *w*Mel; [62–64]), to find similarities with the predicted protein sets of the newly assembled *w*Fex genome. Orthologs were identified using OrthoVenn (E-value cut-off of 10^− 5^ and inflation value 1.5). In addition, we analyzed the paralogous genes within the *w*Fex genome, to help understand the increased genome size in comparison to other *Wolbachia* genomes.

### Discovery and annotation of transposable elements

We used RepeatMasker version 4.0.7 [108], and the TransposonPSI version 08-22-2010 [109] to detect repetitive elements in the genome. To retrieve and mask repetitive elements, we downloaded files from the Repbase and Dfam databases, and aligned each of them with the *F. exsecta* genome sequences as query sequences. Positive alignments were regarded as repetitive regions and extracted for further analysis. To identify genome sequence region homology to proteins encoded by different families of transposable elements, we used the TransposonPSI analysis tool. This tool uses PSI-BLAST, with a collection of retro-transposon ORF homology profiles to identify statistically significant alignments.

### *Wolbachia* phylogeny

We analysed the phylogeny of *Wolbachia* in MrBayes v3.2.6 × 64 [110], using a concatenated sequences of 12 genes which were present as single copy in *w*Fex genome. For this analysis, each gene was considered as a different partition, and the most fitting nucleotide substitution model was chosen for each gene, using the bayesian information criterion (BIC) in the program jMODELTEST (Posada, 2008). The partitioned dataset was run for 200,000 generations, sampling at every 100th generation with each partition unlinked for the substitution parameters. Convergence of the runs was confirmed by checking that the potential scale reduction factor was ~ 1.0 for all model parameters, and by ensuring that an average split frequency of standard deviations <0.01 was reached [110]. The first 25% of the trees were discarded as burn-in, and the remaining trees were used to create a 50% majority-rule consensus tree, and to estimate the posterior probabilities. To check for consistency of the phylogeny, Markov chain Monte Carlo (MCMC) runs were repeated to get a similar 50% majority-rule consensus tree with high posterior probabilities. The phylogenetic tree generated was visualized using Figtree v1.4.2 [111].

## Additional files

Additional file 1: **Table S1.** Comparison of assembly statistics of the *F. exsecta* genome and 13 other published ant genomes. (XLSX 11 kb)

Additional file 2: **Table S2.** List of genes specific to the Formicinae as identified by OrthoVenn. (XLSX 20 kb)

Additional file 3: **Table S3.** List of species-specific genes in *F. exsecta,* as identified by OrthoVenn. (XLSX 24 kb)

Additional file 4: **Table S4.** List of *F. exsecta* genes under positive or relaxed purifying selection (dN/dS ratios > 1) in comparison to five other ant species (*Camponotus floridanus, Lasius niger, Solenopsis invicta* and *Cerapachys biroi*) (XLSX 115 kb)

Additional file 5: **Table S5.** List of *F. exsecta* genes showing dN/dS ratios > 1 in pairwise comparison to *Camponotus floridanus. (XLSX 11 kb)*

Additional file 6: **Figure S1.** Visualization of genome coverage of *w*Fex against the *Wolbachia* endosymbiont of *Drosophila simulans* (*w*Ri) genome, and *Dactylopius coccus* (*w*Dac), using the alignment software circoletto. (PDF 2210 kb)

Additional file 7: **Table S6.** List of genes with paralogs in the *w*Fex genome, which are present as single copies in the *w*Mel, *w*Ri, *w*Dac genomes. (XLSX 27 kb)

Additional file 8: **Table S7.** List of conserved *Wolbachia* genes used for phylogenetic analysis. (XLSX 14 kb)

Additional file 9: **Table S8.** List of species-specific genes in *w*Fex genome*,* as identified by OrthoVenn. (XLSX 15 kb)

Additional file 10: **Figure S2.** Venn diagram displaying the overlap in orthologous genes across CI-inducing and mutualist *Wolbachia* species. (PDF 98 kb)

Additional file 11: **Figure S3.** Schematic representation of *cifA* and *cifB* gene locations on the *w*Fex genome assembly. (PDF 27 kb)

Additional file 12: **Figure S4.** Visualization of sequence similarity between chromosomal *Wolbachia* and cytoplasmic *Wolbachia*, using the alignment software circoletto. (PDF 777 kb)

Additional file 13: **Figure S5.** TAGC plot of *F. exsecta,* and its *Wolbachia* endosymbiont. The TAGC plots were taxonomically annotated, and the contigs with best similarity to Arthropoda and Proteobacteria are highlighted in color. (PDF 121 kb)

## Abbreviations

BP: BasePair
BUSCO: Benchmarking Universal Single-Copy Orthologs
CEGMA: Core Eukaryotic Genes Mapping Approach
COG: Cluster of Orthologous Groups
EC: enzyme commission (EC)
ESTs: Expressed Sequence Tag
FPKM: Fragments Per Kilobase Million
GAGA: Global Ant Genome Alliance
HGT: Horizontal Gene Transfer
LSU rRNA: large subunit ribosomal ribonucleic acid
MB: MegaBases
MCMC: Markov chain Monte Carlo
OGS: Official Gene Set
ORF: Open Reading Frame
SGA: String Graph Assembler
SSU rRNA: Small subunit ribosomal ribonucleic acid
TEs: Transposable Elements
TrEMBL: Translation of EMBL nucleotide sequence database
