# Supplementary material for: The first draft genomes of the ant Formica exsecta, and its Wolbachia endosymbiont reveal extensive gene transfer from endosymbiont to host
Source: BMC Genomics. 2019 Apr 16;20:301. doi: 10.1186/s12864-019-5665-6 (PMC6469114; doi:10.1186/s12864-019-5665-6)
Supplement: Supplementary file 6 — Figure S1. Visualization of genome coverage of wFex against the Wolbachia endosymbiont of Drosophila simulans (wRi) genome, and Dactylopius coccus (wDac), using the alignment software circoletto. (PDF 2210 kb) [file 12864_2019_5665_MOESM6_ESM.pdf]

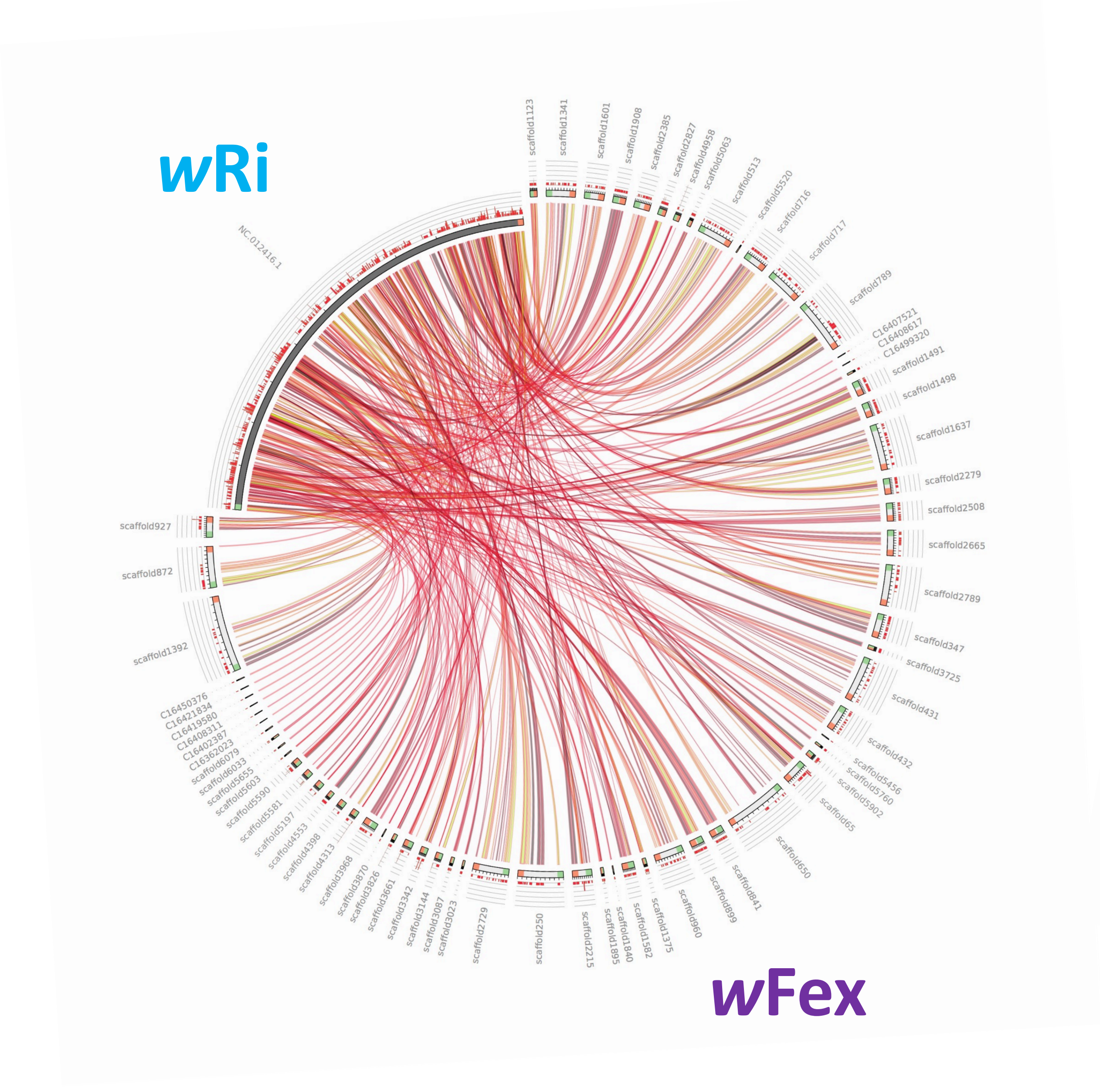
*w*Ri
*w*Fex

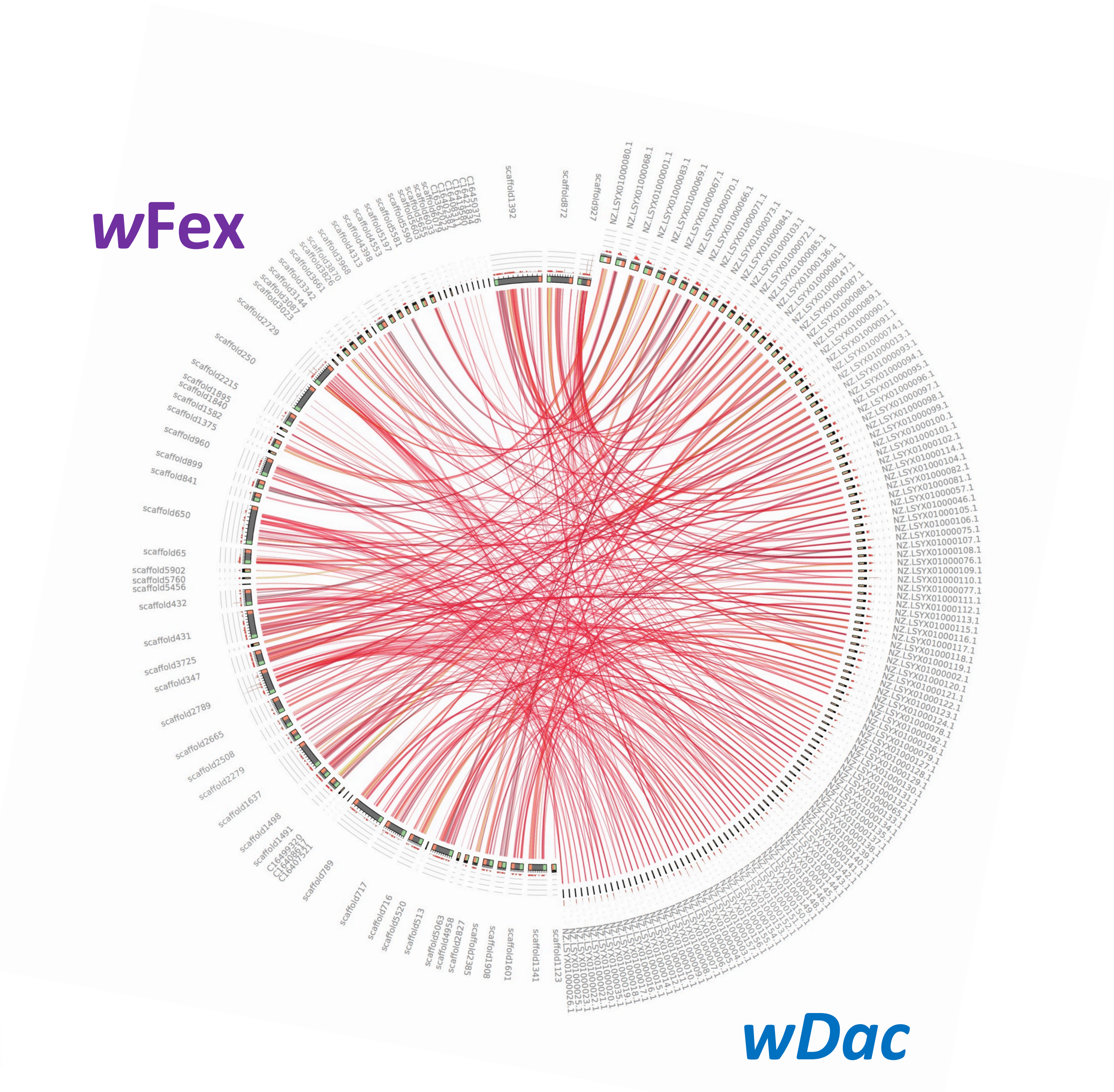
*w*Fex
*w*Dac
