# Supplementary figures and images for: The first draft genomes of the ant Formica exsecta, and its Wolbachia endosymbiont reveal extensive gene transfer from endosymbiont to host
Source: BMC Genomics. 2019 Apr 16;20:301. doi: 10.1186/s12864-019-5665-6 (PMC6469114; doi:10.1186/s12864-019-5665-6)

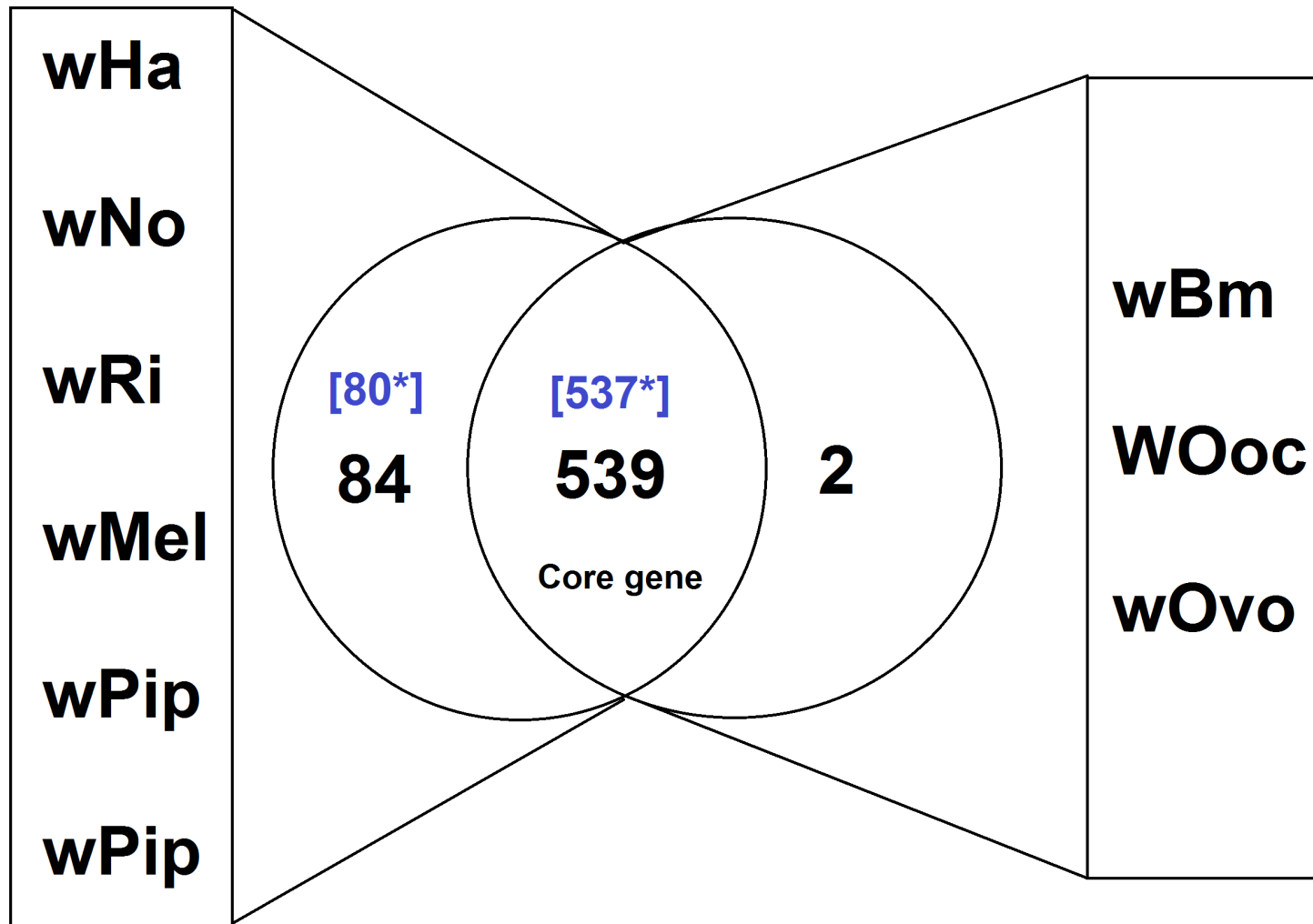

Cytoplasmic Incompatibility (CI)

Mutualist

\* Genes in wFEX Genome

Supplement: Supplementary file 10 — Figure S2. Venn diagram displaying the overlap in orthologous genes across CI-inducing and mutualist Wolbachia species. (PDF 98 kb) [file 12864_2019_5665_MOESM10_ESM.pdf]

Scaffold789:1-163347

NCBI ID : RCIU01000013

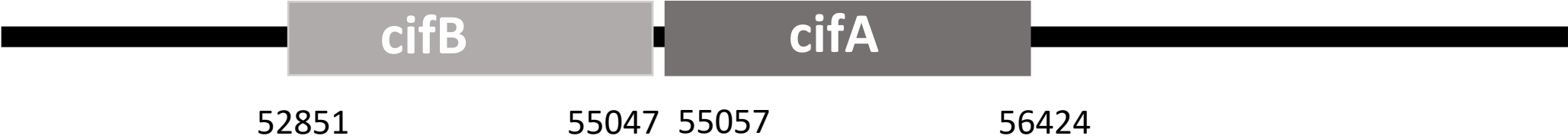

Scaffold650:1-187047

NCBI ID : RCIU01000032

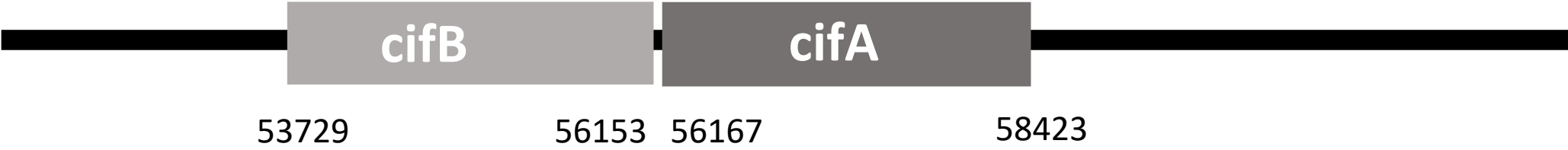

Supplement: Supplementary file 11 — Figure S3. Schematic representation of cifA and cifB gene locations on the wFex genome assembly. (PDF 27 kb) [file 12864_2019_5665_MOESM11_ESM.pdf]

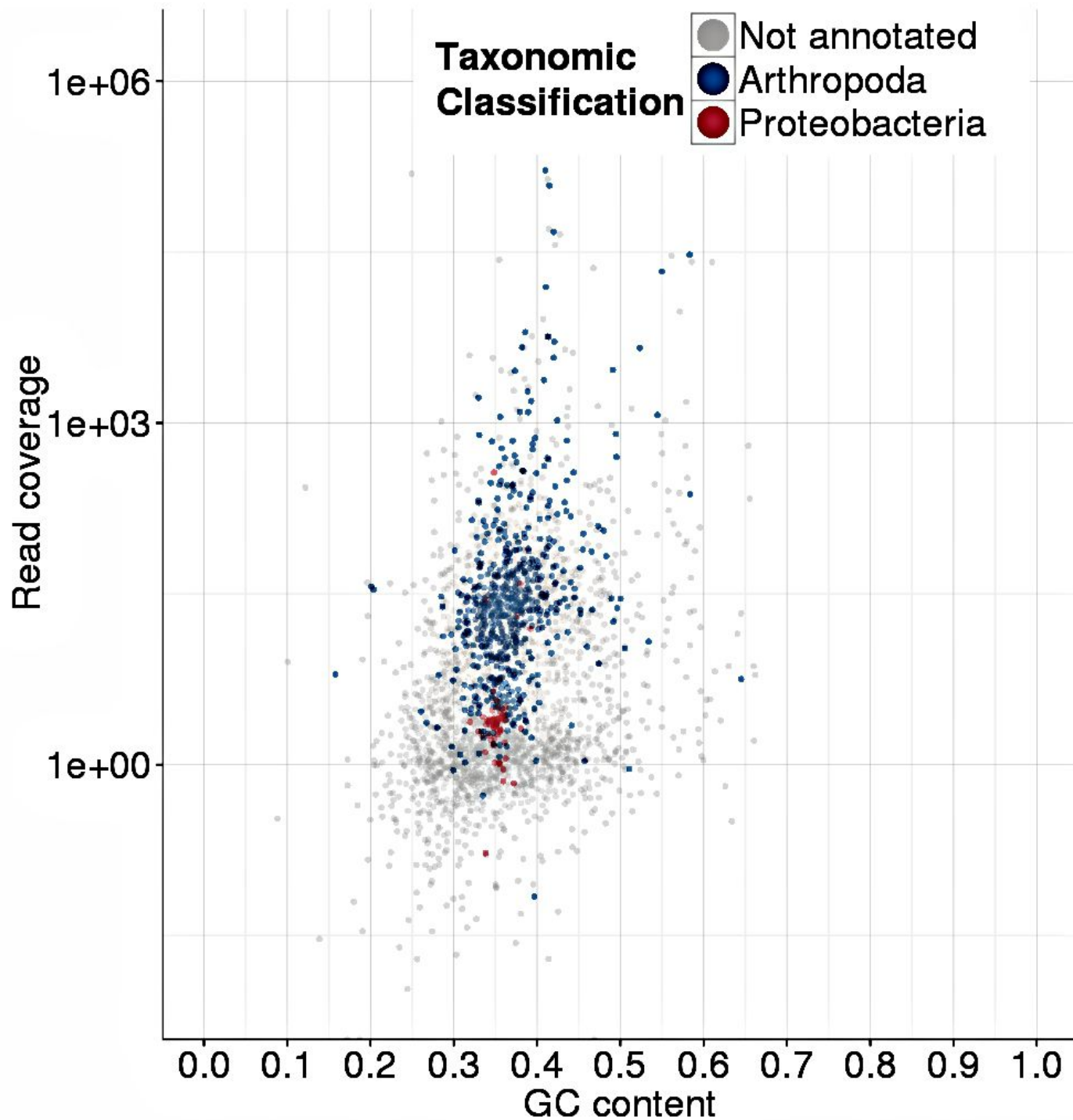

Supplement: Supplementary file 13 — Figure S5. TAGC plot of F. exsecta, and its Wolbachia endosymbiont. The TAGC plots were taxonomically annotated, and the contigs with best similarity to Arthropoda and Proteobacteria are highlighted in color. (PDF 121 kb) [file 12864_2019_5665_MOESM13_ESM.pdf]
