# Supplementary material for: The first draft genomes of the ant Formica exsecta, and its Wolbachia endosymbiont reveal extensive gene transfer from endosymbiont to host
Source: BMC Genomics. 2019 Apr 16;20:301. doi: 10.1186/s12864-019-5665-6 (PMC6469114; doi:10.1186/s12864-019-5665-6)
Supplement: Supplementary file 12 — Figure S4. Visualization of sequence similarity between chromosomal Wolbachia and cytoplasmic Wolbachia, using the alignment software circoletto. (PDF 777 kb) [file 12864_2019_5665_MOESM12_ESM.pdf]

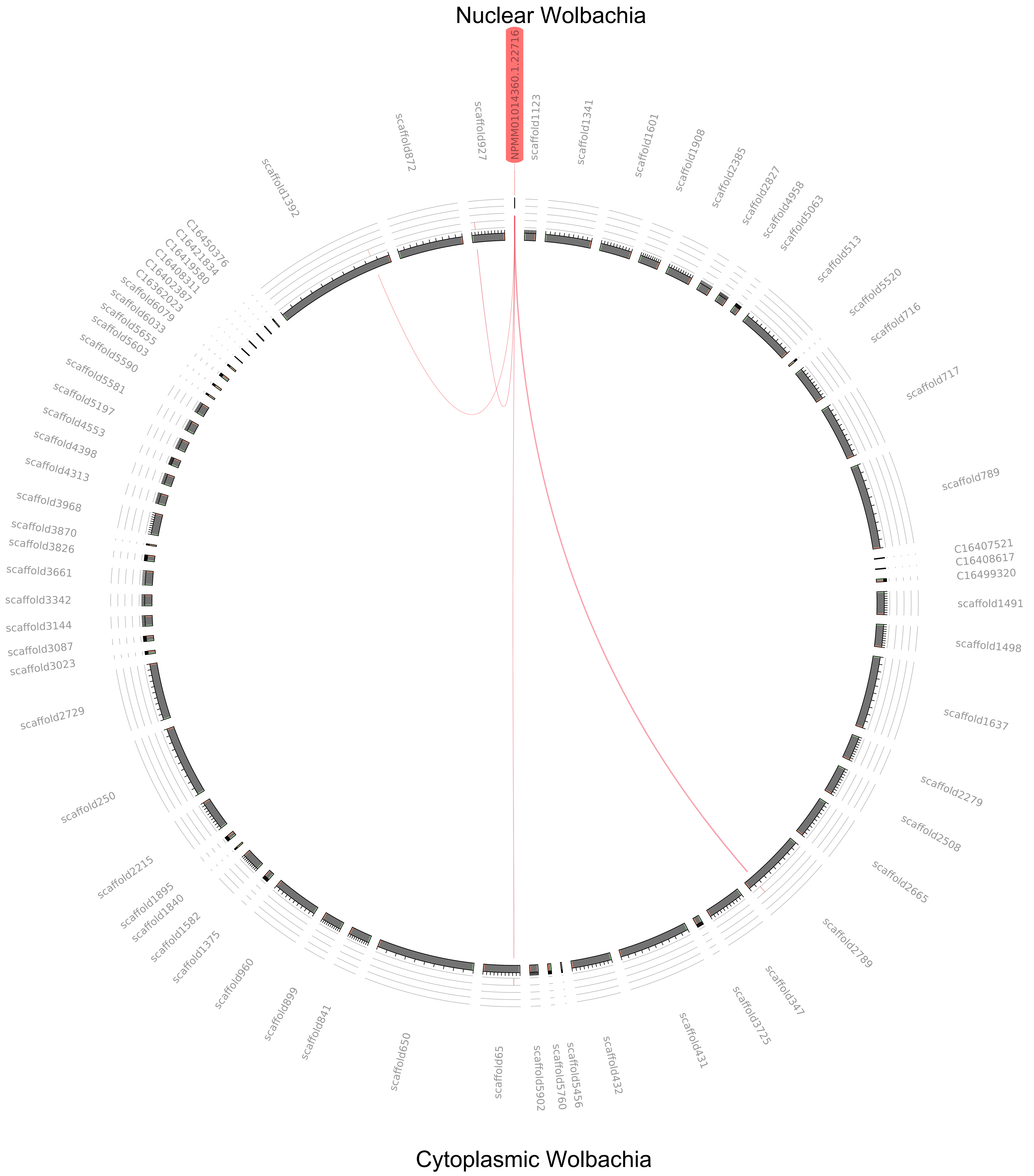
Nuclear Wolbachia
NPMM01014360.1.22716
scaffold1123
scaffold1341
scaffold1601
scaffold1908
scaffold2385
scaffold2827
scaffold4958
scaffold5063
scaffold513
scaffold5520
scaffold716
scaffold717
scaffold789
C16407521
C16408617
C16499320
scaffold1491
scaffold1498
scaffold1637
scaffold2279
scaffold2508
scaffold2665
scaffold2789
scaffold347
scaffold3725
scaffold431
scaffold432
scaffold5456
scaffold5760
scaffold5902
scaffold65
scaffold650
scaffold841
scaffold899
scaffold960
scaffold1375
scaffold1582
scaffold1840
scaffold1895
scaffold2215
scaffold250
scaffold2729
scaffold3023
scaffold3087
scaffold3144
scaffold3342
scaffold3661
scaffold3826
scaffold3870
scaffold3968
scaffold4313
scaffold4398
scaffold4553
scaffold5197
scaffold5581
scaffold5590
scaffold5603
scaffold5655
scaffold6033
scaffold6079
C16362023
C16402387
C16408311
C16419580
C16421834
C16450376
scaffold1392
scaffold872
scaffold927
Cytoplasmic Wolbachia

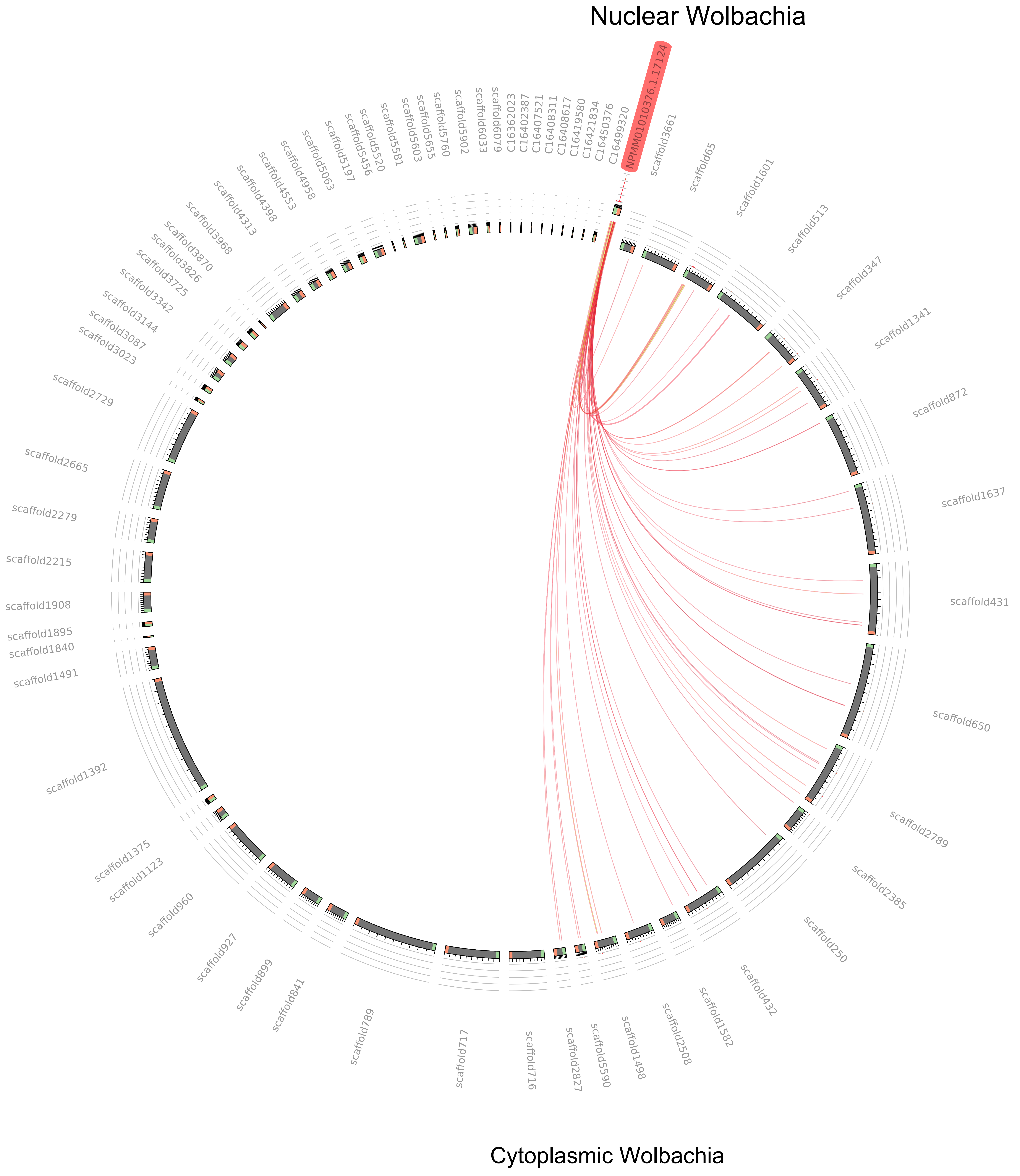
Nuclear Wolbachia
NPMM01010376.1.17124
Cytoplasmic Wolbachia
scaffold3661
scaffold65
scaffold1601
scaffold513
scaffold347
scaffold1341
scaffold872
scaffold1637
scaffold431
scaffold650
scaffold2789
scaffold2385
scaffold250
scaffold432
scaffold1582
scaffold2508
scaffold1498
scaffold5590
scaffold2827
scaffold716
scaffold717
scaffold789
scaffold841
scaffold899
scaffold927
scaffold960
scaffold1123
scaffold1375
scaffold1392
scaffold1491
scaffold1840
scaffold1895
scaffold1908
scaffold2215
scaffold2279
scaffold2665
scaffold2729
scaffold3023
scaffold3087
scaffold3144
scaffold3342
scaffold3725
scaffold3826
scaffold3870
scaffold3968
scaffold4313
scaffold4398
scaffold4553
scaffold4958
scaffold5063
scaffold5197
scaffold5456
scaffold5520
scaffold5581
scaffold5603
scaffold5655
scaffold5760
scaffold5902
scaffold6033
scaffold6079
C16362023
C16402387
C16407521
C16408311
C16408617
C16419580
C16421834
C16450376
C16499320

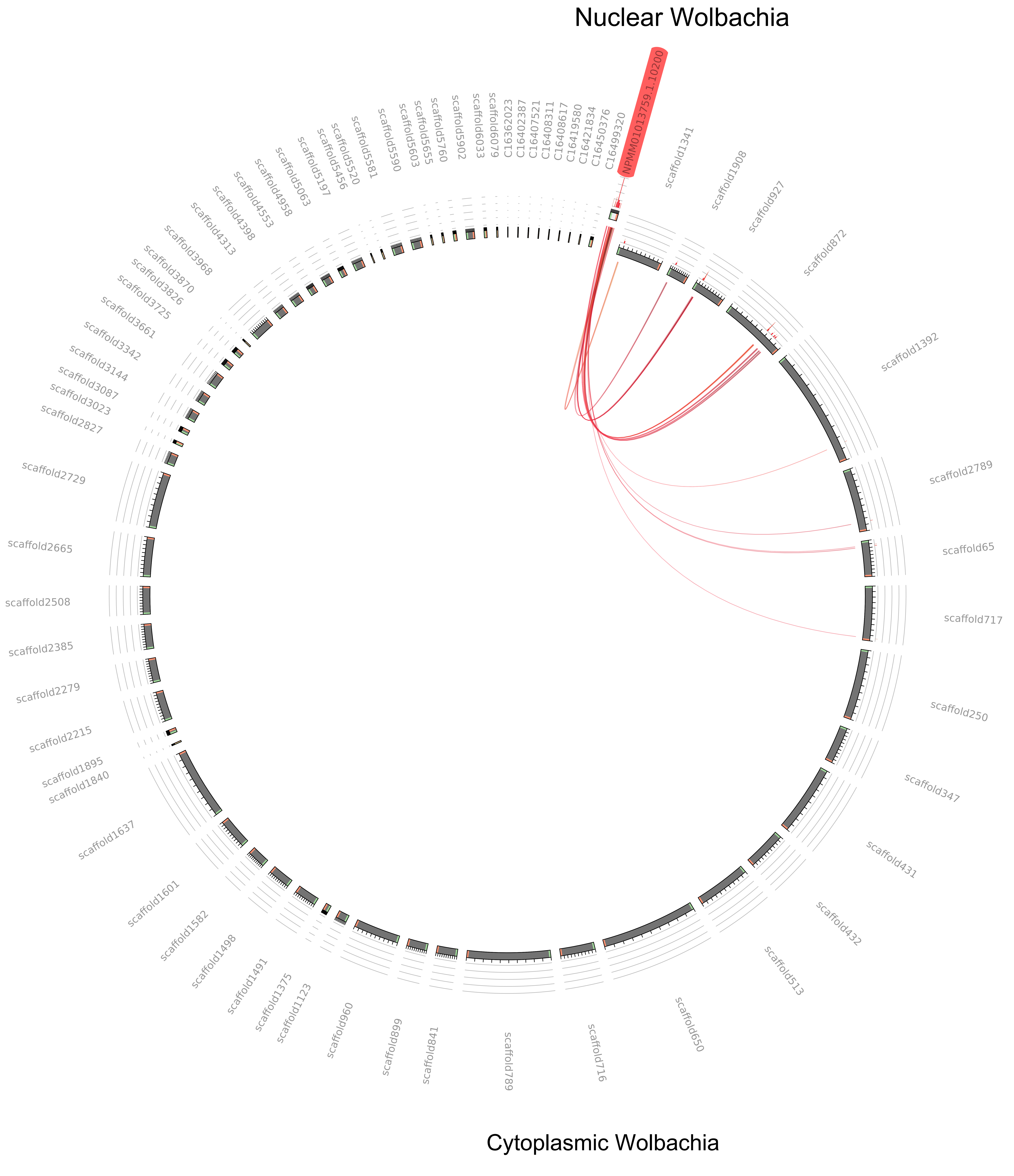
Nuclear Wolbachia
NPMM01013759.1.10200
Cytoplasmic Wolbachia
scaffold1341
scaffold1908
scaffold927
scaffold872
scaffold1392
scaffold2789
scaffold65
scaffold717
scaffold250
scaffold347
scaffold431
scaffold432
scaffold513
scaffold650
scaffold716
scaffold789
scaffold841
scaffold899
scaffold960
scaffold1123
scaffold1375
scaffold1491
scaffold1498
scaffold1582
scaffold1601
scaffold1637
scaffold1840
scaffold1895
scaffold2215
scaffold2279
scaffold2385
scaffold2508
scaffold2665
scaffold2729
scaffold2827
scaffold3023
scaffold3087
scaffold3144
scaffold3342
scaffold3661
scaffold3725
scaffold3826
scaffold3870
scaffold3968
scaffold4313
scaffold4398
scaffold4553
scaffold4958
scaffold5063
scaffold5197
scaffold5456
scaffold5520
scaffold5581
scaffold5590
scaffold5603
scaffold5655
scaffold5760
scaffold5902
scaffold6033
scaffold6079
C16362023
C16402387
C16407521
C16408311
C16408617
C16419580
C16421834
C16450376
C16499320

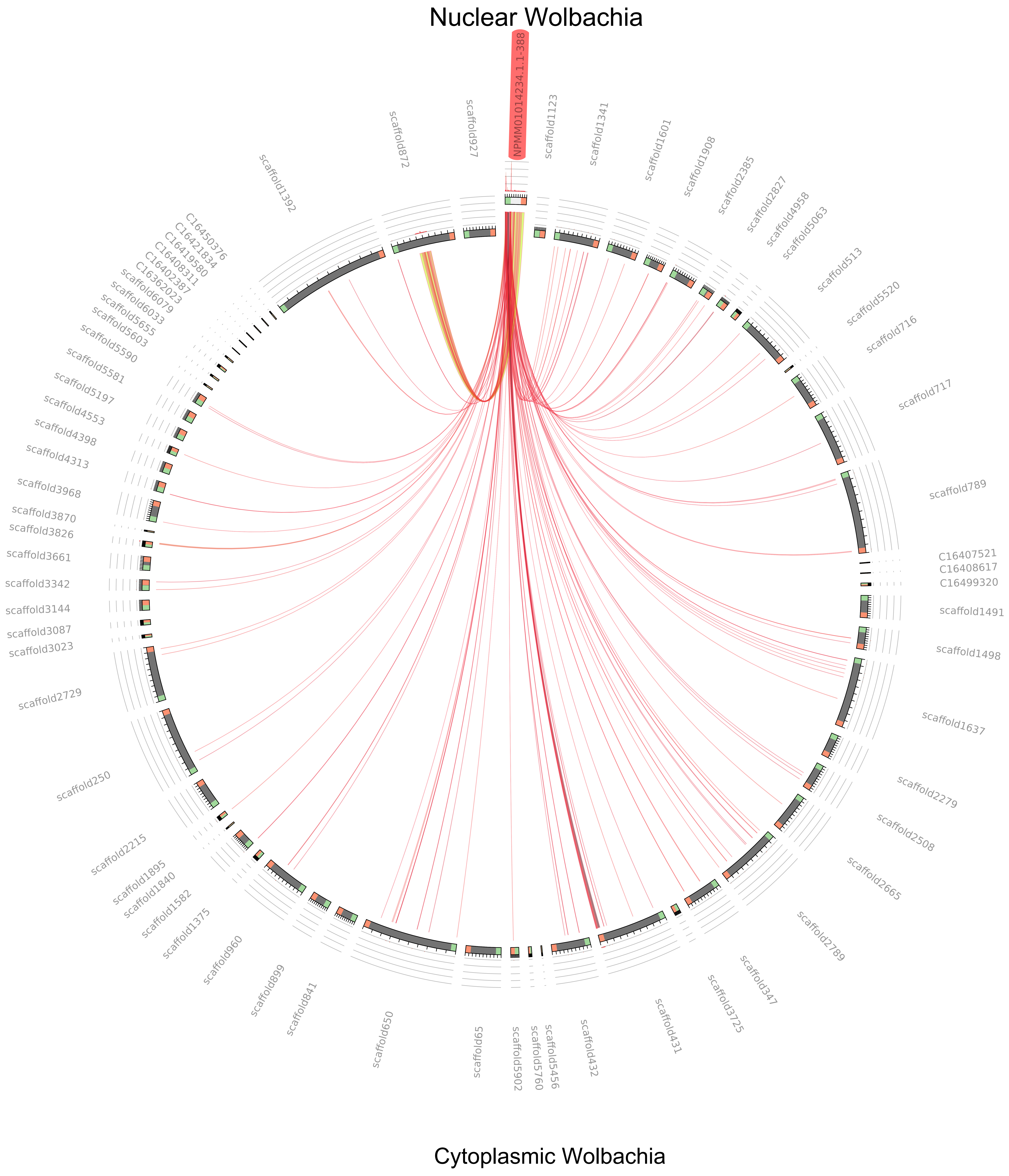
Nuclear Wolbachia
NPMM01014234.1.1-388
scaffold927
scaffold872
scaffold1392
scaffold1123
scaffold1341
scaffold1601
scaffold1908
scaffold2385
scaffold2827
scaffold4958
scaffold5063
scaffold513
scaffold5520
scaffold716
scaffold717
scaffold789
C16407521
C16408617
C16499320
scaffold1491
scaffold1498
scaffold1637
scaffold2279
scaffold2508
scaffold2665
scaffold2789
scaffold347
scaffold3725
scaffold431
scaffold432
scaffold5456
scaffold5760
scaffold5902
scaffold65
scaffold650
scaffold841
scaffold899
scaffold960
scaffold1375
scaffold1582
scaffold1840
scaffold1895
scaffold2215
scaffold250
scaffold2729
scaffold3023
scaffold3087
scaffold3144
scaffold3342
scaffold3661
scaffold3826
scaffold3870
scaffold3968
scaffold4313
scaffold4398
scaffold4553
scaffold5197
scaffold5581
scaffold5590
scaffold5603
scaffold5655
scaffold6033
scaffold6079
C16362023
C16402387
C16408311
C16419580
C16421834
C16450376
Cytoplasmic Wolbachia

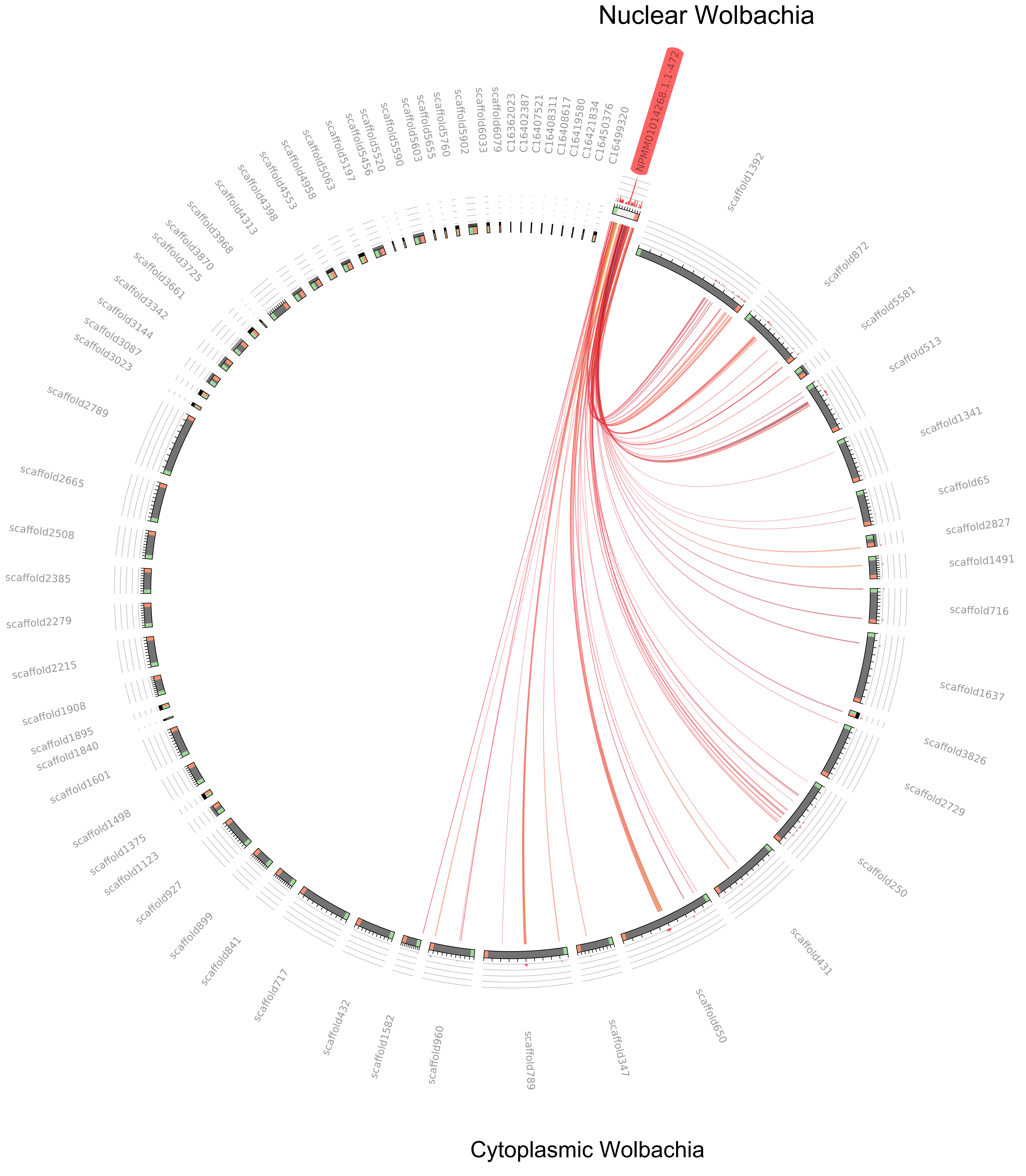
Nuclear Wolbachia
NPMM01014268.1.1-472
scaffold1392
scaffold872
scaffold5581
scaffold513
scaffold1341
scaffold65
scaffold2827
scaffold1491
scaffold716
scaffold1637
scaffold3826
scaffold2729
scaffold250
scaffold431
scaffold650
scaffold347
scaffold789
scaffold960
scaffold1582
scaffold432
scaffold717
scaffold841
scaffold899
scaffold927
scaffold1123
scaffold1375
scaffold1498
scaffold1601
scaffold1840
scaffold1895
scaffold1908
scaffold2215
scaffold2279
scaffold2385
scaffold2508
scaffold2665
scaffold2789
scaffold3023
scaffold3087
scaffold3144
scaffold3342
scaffold3661
scaffold3725
scaffold3870
scaffold3968
scaffold4313
scaffold4398
scaffold4553
scaffold4958
scaffold5063
scaffold5197
scaffold5456
scaffold5520
scaffold5590
scaffold5603
scaffold5655
scaffold5760
scaffold5902
scaffold6033
scaffold6079
C16362023
C16402387
C16407521
C16408311
C16408617
C16419580
C16421834
C16450376
C16499320
Cytoplasmic Wolbachia
